# Intermittent Screening and Treatment versus Intermittent Preventive Treatment of Malaria in Pregnancy: Provider Knowledge and Acceptability

**DOI:** 10.1371/journal.pone.0024035

**Published:** 2011-08-24

**Authors:** Lucy Smith Paintain, Gifty D. Antwi, Caroline Jones, Esther Amoako, Rose O. Adjei, Nana A. Afrah, Brian Greenwood, Daniel Chandramohan, Harry Tagbor, Jayne Webster

**Affiliations:** 1 Department of Disease Control, Faculty of Infectious and Tropical Diseases, London School of Hygiene and Tropical Medicine, London, United Kingdom; 2 Department of Community Health, School of Medical Sciences, Kwame Nkrumah University of Science and Technology, Kumasi, Ghana; Laboratory of Malaria Immunology and Vaccinology, United States of America

## Abstract

Malaria in pregnancy (MiP) is associated with increased risks of maternal and foetal complications. The WHO recommends a package of interventions including intermittent preventive treatment (IPT) with sulphadoxine-pyrimethamine (SP), insecticide-treated nets and effective case management. However, with increasing SP resistance, the effectiveness of SP-IPT has been questioned. Intermittent screening and treatment (IST) has recently been shown in Ghana to be as efficacious as SP-IPT. This study investigates two important requirements for effective delivery of IST and SP-IPT: antenatal care (ANC) provider knowledge, and acceptance of the different strategies. Structured interviews with 134 ANC providers at 67 public health facilities in Ashanti Region, Ghana collected information on knowledge of the risks and preventative and curative interventions against MiP. Composite indicators of knowledge of SP-IPT, and case management of MiP were developed. Log binomial regression of predictors of provider knowledge was explored. Qualitative data were collected through in-depth interviews with fourteen ANC providers with some knowledge of IST to gain an indication of the factors influencing acceptance of the IST approach. 88.1% of providers knew all elements of the SP-IPT policy, compared to 20.1% and 41.8% who knew the treatment policy for malaria in the first or second/third trimesters, respectively. Workshop attendance was a univariate predictor of each knowledge indicator. Qualitative findings suggest preference for prevention over cure, and increased workload may be barriers to IST implementation. However, a change in strategy in the face of SP resistance is likely to be supported; health of pregnant women is a strong motivation for ANC provider practice. If IST was to be introduced as part of routine ANC activities, attention would need to be given to improving the knowledge and practices of ANC staff in relation to appropriate treatment of MiP. Health worker support for any MiP intervention delivered through ANC clinics is critical.

## Introduction

*Plasmodium falciparum* infection in pregnancy is associated with an increased risk of maternal and foetal complications including maternal anaemia and low birth weight [1], [2]. The WHO has recommended a package of interventions for preventing and controlling malaria infection in pregnancy (MiP) in endemic areas, which includes the early diagnosis and treatment of malaria, the use of insecticide-treated nets (ITNs), and intermittent preventive treatment during pregnancy (IPT) using sulphadoxine-pyrimethamine (SP)[3].

However, in recent years resistance to SP has spread across sub-Saharan Africa and thus the effectiveness of SP-IPT has been questioned [4]. There are few anti-malarial drugs with plasma half-life comparable to that of SP that could be used to replace SP for IPT in areas with high SP resistance. Furthermore, there is very limited information on the efficacy and safety during pregnancy of alternative drugs [5]. A three-arm, open label, individually-randomized trial was conducted in the Ashanti Region of Ghana between February 2007 and November 2008 to investigate the efficacy of an alternative strategy: in two of the arms, pregnant women were screened at scheduled antenatal care (ANC) visits with a rapid diagnostic test (RDT) and those with a positive result were treated with either artesunate-amodiaquine (AS–AQ) or SP; women in the third arm received three doses of SP-IPT at monthly intervals as per current national policy. The alternative strategy, termed intermittent screening and treatment (IST) was found to be non-inferior to SP-IPT in preventing maternal anaemia and low birth weight [6].

Although IST appears to be a promising alternative to SP-IPT for the control of malaria in pregnancy, translating efficacy from a controlled trial setting to effectiveness under operational conditions is challenging [7]. ANC attendance is very high in Ashanti and many parts of sub-Saharan Africa, suggesting that delivery of MiP interventions through this channel would achieve high coverage. However, acceptability of the intervention by both users and providers is crucial, as well as issues of cost effectiveness and capacity for implementation within the existing health system.

Despite the fact that 36 malaria-endemic countries have adopted SP-IPTp as national policy [8], there is little literature on the malaria-related knowledge of ANC staff or their practices [9]. Existing perceptions, levels of knowledge and experience will influence how a new strategy such as IST is accepted and adopted by those who will be implementing it. For example, if a policy of IST was to be implemented by ANC providers, it is assumed that they would need to be trained in the use and interpretation of RDTs. However, it may also be necessary to include additional malaria in pregnancy topics such as prescribing and dispensing of treatments, to support effective IST implementation.

An investigation of the perceptions and experiences of pregnant women participating in the Ashanti IST trial showed that, overall, the two strategies were equally acceptable and other factors such as quality of care and health benefits for themselves and their babies were perceived to be more important elements in affecting the acceptability of ANC strategies [10].

Here we present the perspective of providers of ANC in the public sector of Ashanti Region, in relation to their knowledge of malaria in pregnancy and how it can be treated or prevented.

## Methods

### Ethics statement

Ethical approval for this study was granted by the Committee on Human Research, Publications and Ethics, Kwame Nkrumah University of Science and Technology, School of Medical Sciences, Kumasi, Ghana and by the ethics committee of the London School of Hygiene & Tropical Medicine. Written informed consent was sought from all participants before the start of all interviews (structured, or semi-structured), including permission to use anonymous quotes. During transcription of recorded key informant interviews, any names were replaced with codes to ensure anonymity and digital recordings were deleted once transcription was completed and quality approved.

### Study area

The Ashanti Region of Ghana has 27 districts with a total population of approximately 3.61 million. Ashanti is predominantly rural. Transmission of malaria is moderately high [11] and occurs throughout the year with peaks during the rains from May-October.

In 2008, 97.0% of women in the Ashanti Region received ANC from a skilled provider at least once during their most recent pregnancy [12]. All health facilities with an ANC clinic in the study districts offer SP-IPT to pregnant women following the Ministry of Health (MoH) guidelines which recommend three doses of SP-IPT. According to the national guidelines, pregnant women in their first trimester diagnosed with malaria should be treated with quinine; those diagnosed with malaria in their second or third trimester should be treated with AS-AQ. There is no policy on screening pregnant women for malaria on attendance at the ANC clinic. Counselling and testing for prevention of mother to child transmission of HIV (PMTCT) is also part of core ANC services in Ghana, with HIV prevalence amongst adults aged 15–49 in Ghana at 1.8% [13]. All maternal health care, including delivery has been free since July 2008, a policy implemented through the National Health Insurance Scheme.

### Health facility audit & health worker survey

The sampling scheme was designed to be representative of ANC providers at the regional level. Based upon feasibility within existing resources, 7 of 21 districts were randomly selected. Although the number of districts in Ashanti had been recently expanded, population data taking this into account were not available at the time of the study. Therefore, selection was based on the 21 districts as defined at the time of the 2000 census. Kumasi Metropolitan district was purposively sampled because it has a catchment population of approximately one-third of the regional population. Ejisu-Juaben and Sekyere East districts were excluded from the sampling frame to avoid facilities that had been involved in the Ashanti IST trial as their ANC staff may not be representative of the region as a whole. The remaining six districts were randomly selected from the sampling frame of 18 districts using population proportional to size.

All public and mission health facilities in these seven districts (Adansi South, Afigya-Sekyere, Amansie West, Asante Akim South, Bosomtwi/Atwima/Kwanhuma, Kumasi Metropolitan, Offinso) that provide antenatal services were included.

Structured audits of each of the sampled health facilities were conducted to investigate the personnel profile, laboratory and dispensing services available at the facility, and availability of anti-malarial drugs and rapid diagnostic tests in the facility and at the ANC clinic.

All staff in the ANC clinic present on the day of the visit were interviewed individually using a structured questionnaire, including questions on health worker demographics, responsibilities in the ANC clinic, recent training on malaria in pregnancy, knowledge of the risks and consequences of malaria in pregnancy, knowledge of the national policies for IPT and treatment of uncomplicated malaria in pregnancy.

Facility audits and structured interviews with ANC staff were conducted between August and December 2009. Data were double-entered and validated in EpiData version 3.1 [14]. Stata 11.0 [15] was used for data processing and analysis. All analyses adjusted for clustering at the health facility level.

Composite indicators were constructed based on full knowledge of all components of current national policy for IPT and treatment of malaria in pregnancy as follows: (i) full knowledge of IPTp was defined as the proportion of ANC providers who knew the correct drug, dose and timing, that use of SP-IPT is restricted in the ninth month and that SP-IPT should be administered as directly-observed therapy (DOT); (ii) full knowledge of treatment for uncomplicated malaria in the first trimester of pregnancy was defined as the proportion of ANC providers who knew the correct drug (quinine), dose and duration; (iii) full knowledge of treatment for uncomplicated malaria in the second and third trimesters of pregnancy was defined as the proportion of ANC providers who knew the correct drug (AS–AQ), dose and duration, including restrictions on use of AS–AQ during first trimester.

Potential determinants of each of these three composite IPT and treatment knowledge indicators were explored using log binomial regression, adjusted for cluster effect at the facility level. Where appropriate, univariate predictors with p-values of <0.1 were included in a multivariate model.

### Key informant interviews

In-depth key informant interviews were conducted in January and February 2009 in Ejisu-Juaben and Sekyere East districts with a total of fourteen midwives and nursing assistants providing routine ANC in the six facilities where the Ashanti IST trial took place. As many providers as possible in each ANC clinic were sought for interview and data collection continued until no new themes were emerging. Interview topic guides were used, covering provider perceptions and experience of the risks and consequences of malaria in pregnancy, experience and opinions of implementing SP-IPT, prior experience of conducting blood tests and their opinions on the IST strategy under investigation at their health facilities.

All interviews were conducted in English after checking that the interviewee was comfortable expressing their opinions in this language. With the informed consent of the participants, all interviews were digitally recorded. Transcriptions were coded and analysed by one of the authors (LSP) using NVivo version 8 [16]. A content analysis approach was used to identify themes based around the providers' opinions and experiences of malaria in pregnancy [17]. The qualitative findings are used here to provide an indication of provider acceptability of the IST approach and to complement the quantitative findings of the health worker survey.

## Results

### Health facility audit

Sixty-seven ANC-providing health facilities in 7 districts of the Ashanti Region were surveyed between August and December 2009. Of these 67 facilities, 77.6% (52) were government-run and the remainder were mission facilities. Just over half were health centres (50.8%, 34/67), 22.4% (15) were hospitals, 19.4% (13) rural health clinics and 7.5% (5) community clinics. The median number of new ANC registrants in 2008 across all facilities was 367 (interquartile range, IQR: 178–774) and the median total number of ANC attendants in 2008 was 1136 (IQR: 434–2334). In both cases, the data are highly skewed as the hospitals had considerably higher ANC caseloads with a median of 1644 new ANC registrants; two hospitals had over 5000 new registrants in 2008.

Almost all of the surveyed facilities had a drug dispensary (91.0%, 61/67). The majority (70.1%, 47/67) kept SP in the ANC for dispensing by midwives; of those that did not, 45.0% (9/20) were health centres, 25.0% (5/20) rural health clinics, 15.0% (3/20) hospitals and 15.0% (3/20) community clinics. Considerably fewer facilities (25.4%, 17/67) kept AS–AQ available in the ANC for dispensing by midwives; of those that did 64.7% (11/17) were health centres, 23.5% (4/17) were rural health clinics and 11.8% (2/17) were community clinics; none of the hospitals dispensed AS–AQ in their ANC clinics (Table 1).

**Table 1:** Characteristics of the health facilities that participated in the study, according to level of facility.

		Hospital(N = 15)	Health centre (N = 34)	Rural health clinic (N = 13)	Community clinic(N = 5)	Total(N = 67)
		n (%)	n (%)	n (%)	n (%)	n (%)
Dispensary in facility	Yes	15 (100)	32 (94.1)	10 (76.9)	4 (80.0)	61 (91.0)
	No	0	2 (5.9)	3 (23.1)	1 (20.0)	6 (9.0)
Drugs dispensed outside dispensary*	No	4 (26.7)	21 (61.8)	7 (53.9)	3 (60.0)	35 (52.2)
	Inpatients	8 (53.3)	2 (5.9)	0	0	10 (14.9)
	Outpatients	2 (13.3)	2 (5.9)	4 (30.8)	1 (20.0)	9 (13.4)
	ANC	9 (60.0)	13 (38.2)	5 (38.5)	1 (20.0)	28 (41.8)
SP kept in ANC for use by midwives	Yes	12 (80.0)	25 (73.5)	8 (61.5)	2 (40.0)	47 (70.2)
	No	3 (20.0)	9 (26.5)	5 (61.5)	3 (60.0)	20 (29.8)
AS–AQ kept in ANC for use by midwives	Yes	0	11 (32.4)	4 (30.8)	2 (40.0)	17 (25.4)
	No	15 (100)	23 (67.7)	9 (69.2)	3 (60.0)	50 (74.6)
Laboratory services provided	Yes	15 (100)	11 (32.4)	3 (23.1)	3 (60.0)	32 (47.8)
	No	0	23 (67.7)	10 (76.9)	2 (40.0)	35 (52.2)
Where laboratory services provided*	On-site	15 (100)	8 (23.5)	3 (23.1)	1 (20.0)	27 (40.3)
	Affiliated external lab	4 (26.7)	4 (11.8)	0	2 (40.0)	10 (14.9)
	ANC	1 (6.7)	1 (2.9)	0	0	2 (3.0)
Functional microscope present	Yes	15 (100)	8 (23.5)	3 (23.1)	1 (20.0)	27 (40.3)
	No	0	26 (76.5)	10 (76.9)	4 (80.0)	40 (59.7)
Malaria blood films conducted	Yes	15 (100)	8 (23.5)	3 (23.1)	1 (20.0)	27 (40.3)
	No	0	26 (76.5)	10 (76.9)	4 (80.0)	40 (59.7)
Malaria RDTs conducted	Yes	7 (46.7)	6 (17.7)	4 (30.8)	0	17 (25.4)
	No	8 (53.3)	23 (67.7)	5 (38.5)	5 (100)	41 (61.2)
	Don't know	0	5 (14.7)	4 (30.8)	0	9 (13.4)
Where malaria RDTs used	Laboratory	7 (46.7)	4 (11.8)	1 (7.7)	0	12 (17.9)
	Outpatients	1 (6.7)	2 (5.9)	3 (23.1)	0	6 (9.0)
	ANC	0	2 (5.9)	2 (15.4)	0	4 (6.0)

*Note: Not mutually exclusive as multiple responses possible.

Just under half of the facilities surveyed had laboratory services (47.8%, 32/67). Around 40% (27/67) had at least one functional microscope and conducted microscopy for malaria parasites. Only 25.4% (17) of facilities currently use malaria RDTs and even fewer (6.0%, 4/67) use RDTs in the ANC clinic (Table 1). Amongst hospitals, 46.7% (7/15) used RDTs, compared to 17.7% of health centres and 30.8% of rural health clinics; none of the community clinics used RDTs. Of the 4 facilities where RDTs were used in the ANC clinic, 2 were rural health clinics and 2 were health centres; none of the hospitals or community clinics used RDTs in the ANC clinic.

### Health worker survey

#### Health worker characteristics

Structured interviews were conducted with 134 health workers working in the 67 public sector antenatal clinics between August and December 2009. On average two health workers were interviewed in each facility (ranging from 1 to 7). The vast majority of ANC personnel interviewed were female (97.8%, 131/134). The median age of respondents was 48 years (IQR: 30–53 years). Half of the respondents were qualified midwives or nurses (50.0%), followed by Community Health Nurses (23.1%), or Nursing or Ward Assistants (22.4%). A minority held other ranks, including orderly, traditional birth attendant, nursing officer or public health nurse (4.5%). Almost half of the respondents had been at the current facility for over 3 years; the proportion that had been in the ANC at the present facility for over 3 years was slightly less at around 40% (Table 2).

**Table 2:** Health worker characteristics.

Variable		N	%
Age (years)	20–29	32	23.9
	30–39	14	10.5
	40–49	32	23.9
	50 and above	56	41.8
Gender	Female	131	97.8
	Male	3	2.2
Cadre	Midwife/Nurse	67	50.0
	Nursing assistant	30	22.4
	Community health nurse	31	23.1
	Other	6	4.5
Time at current facility	Less than 1 year	32	23.9
	1–3 years	36	26.9
	More than 3 years	66	49.3
Time in ANC at current facility	Less than 1 year	38	28.4
	1–3 years	40	29.9
	4–9 years	40	29.9
	10 years or longer	16	11.9
Responsible for dispensing SP-IPT during ANC clinics	Yes	119	88.8
	No	15	11.2
Responsible for prescriptions during ANC clinics	Yes	118	88.1
	No	16	11.9

Around 75% (98/134) of the respondents had attended a workshop on malaria treatment or diagnosis; just over half of these (54/98) were in the same year as the survey or during the previous year. Almost 60% (78/134) of respondents had attended a workshop specifically relating to malaria in pregnancy; 44.9% of these within the last year and an additional 26.9% in the previous year.

#### Knowledge of the consequences of malaria in pregnancy

The health workers were asked their views on the primary causes of anaemia, low birth weight and premature birth. Malaria was the most frequently reported cause of maternal anaemia (79.9%, 107/134), low birth weight (70.2%, 94/134) and premature birth (82.1%, 110/134). Poor diet was also commonly reported to contribute to the same health outcomes (82.1%, 60.5% and 20.9% for maternal anaemia, low birth weight and premature birth, respectively). Anaemia was reported to be an important cause of low birth weight by 50.0% of respondents, and a cause of premature delivery by 47.0%. Other responses on the cause of maternal anaemia, low birth weight and premature delivery included other infections such as sexually-transmitted infections or worms (in the case of anaemia).

All respondents knew at least one consequence of malaria in pregnancy for the mother and baby. The three most common responses to specific questions on the consequences of malaria in pregnancy for the baby were low birth weight (76.1%), premature birth (47.8%) and death *in utero*/abortion (47.0%). For the mother, the three most commonly reported consequences were anaemia (72.4%), weakness (35.1%) and loss of appetite (29.1%); 28.4% reported that the mother might die.

#### Knowledge of national policy for preventive treatment of malaria in pregnancy (IPTp)

Knowledge on most individual components of the national policy for intermittent preventive treatment of malaria in pregnancy was very high; for example only one respondent could not name SP as the drug used for IPT and did not know that women should receive three doses during pregnancy. Ninety-two percent knew that the first dose should be given after the first trimester, 97.8% knew that there should be a minimum of one month between doses, and 96.3% reported that SP-IPT should be administered as directly observed therapy (DOT) (Table 3). When all of these components were combined, 88.1% (118/134) of ANC providers could correctly recall all of the information requested.

**Table 3:** Health worker knowledge of policy for intermittent preventive treatment of malaria in pregnancy (IPTp) in Ashanti Region, Ghana (N = 134 health workers).

Indicator	N	%	95% CI
Proportion who know that SP is correct drug for IPTp	133	99.3	94.7, 99.9
Proportion who know the correct timing of first dose of SP-IPT (after first trimester/16 weeks/quickening)	123	91.8	85.8, 95.4
Proportion who know pregnant women should receive 3 doses of SP-IPT	132	98.5	94.1, 99.6
Proportion who know there should be a minimum of 1 month between SP-IPT doses	131	97.8	93.2, 99.3
Proportion who reported SP-IPT should not be given in the first trimester	70	52.2	43.7, 60.6
Proportion who reported SP-IPT should not be given in the ninth month of pregnancy	73	54.5	45.7, 62.9
Proportion who know that SP-IPT should not be given if a woman is taking cotrimoxazole	0	0	-
Proportion reporting SP-IPT should be administered as directly observed treatment (DOT)	129	96.3	90.2, 98.6
Proportion who know correct drug, dose & timing for SP-IPT and report DOT	118	88.1	81.5, 92.5
Proportion who know correct drug, dose, timing & restriction for SP-IPT (including ninth month but excluding first trimester & cotrimoxazole), and report DOT	64	47.8	39.0, 56.7

The proportion of providers who knew the restrictions on the use of SP-IPT was considerably lower: 52.2% could specify that SP-IPT should not be given in the first trimester, and 54.5% that it should not be given in the ninth month; no respondent suggested existing treatment with cotrimoxazole as a contraindication (Table 3). Combining all elements of the SP-IPT policy, including restriction of SP use in the ninth month, 47.8% (64/134) of ANC providers had full knowledge of SP-IPT administration; if the first trimester timing restriction on the use of SP in pregnancy is also included, then this falls to 15.7% (21/134).

Univariate log binomial regression was conducted to investigate the predictors of ANC providers knowing all aspects of the SP-IPT policy indicator, defined as the proportion who knew correct drug, dose, timing and restrictions for SP-IPT (including ninth month but excluding cotrimoxazole), and reported DOT. The first trimester restriction was not included in the composite indicator as this is already indirectly included by knowledge of the timing of first SP-IPT dose.

Age, cadre, time at health facility and time in the present ANC clinic showed no association with knowledge of the SP-IPT policy. Those responsible for giving SP-IPT during ANC clinic days were almost twice as likely to have full knowledge of the policy (RR: 1.90; 95% CI: 0.87,4.10; p = 0.10) (Table 4).

**Table 4:** Univariate predictors of composite SP-IPT knowledge indicator (N = 134 health workers).

		Knows full SP-IPT policy*			
Predictor	Sub-category	%	RR	95% CI	P value
Age (years)	20–29	62.5	1.00		0.14
	30–39	35.7	0.57	0.26,1.24	
	40–49	40.6	0.65	0.41,1.04	
	50 or older	46.4	0.74	0.51,1.09	
Cadre	Midwife/nurse	49.3	1.00		0.95
	Nursing assistant	43.3	0.88	0.58,1.34	
	Community health nurse	48.4	0.98	0.64,1.51	
	Other	50.0	1.02	0.53,1.92	
Time at health facility	<1 year	53.1	1.00		0.52
	1–3 years	52.8	0.99	0.66,1.50	
	>3 years	42.4	0.80	0.52,1.23	
Time in current ANC	<1 year	55.3	1.00		0.33
	1–3 years	55.0	1.00	0.68,1.45	
	4–9 years	35.0	0.63	0.38,1.06	
	>10 years	43.8	0.79	0.44,1.44	
Responsible for SP-IPT	No	26.7	1.00		0.10
	Yes	50.4	1.90	0.87,4.10	
Attended malaria diagnosis workshop	No	50.0	1.00		0.76
	Yes	46.9	0.94	0.63,1.40	
Year of malaria diagnosis workshop	2009	56.8	1.00		0.04
	2008	58.8	1.04	0.61,1.75	
	2007 or before	34.1	0.60	0.36,1.00	
Attended MiP workshop	No	37.5	1.00		0.05
	Yes	55.1	1.47	1.00,2.16	
Year of MiP workshop	2009	57.1	1.00		0.05
	2008	71.4	1.25	0.87,1.80	
	2007 or before	36.4	0.64	0.34,1.21	

*Defined as the proportion that know the correct drug, dose, timing & restriction for SP-IPT (including ninth month but excluding first trimester and cotrimoxazole), and report DOT.

Recent attendance at a workshop on malaria diagnosis and treatment or at a workshop on malaria in pregnancy (MiP) increased the likelihood that the provider had full knowledge of SP-IPT. For example those that attended a malaria diagnosis workshop in 2007 or earlier were 0.60 times as likely to know about SP-IPT than those who attended a workshop in 2009 (95% CI: 0.36,1.00; p = 0.04); similarly, those who attended a MiP workshop were approximately 1.5 times as likely to know about SP-IPT than those who had not (RR: 1.47; 95% CI: 1.00,2.14; p = 0.05); those who had attended a MiP workshop in 2007 were 0.64 times as likely to know about SP-IPT than those who attended in 2009 (95% CI: 0.34,1.21; p = 0.05) (Table 4).

Given that none of the potential predictors of knowledge except attendance of a MiP workshop were associated with the composite knowledge indicator in the univariate regression, multivariate modelling was not carried out.

#### Knowledge of national policy for treatment of uncomplicated malaria in pregnancy

When asked about the recommended treatment for uncomplicated malaria in the first trimester of pregnancy, only 50.8% (68/134) of ANC providers correctly responded with quinine; 20.2% (27/134) responded with artesunate-amodiaquine (AS–AQ), 14.2% (19/134) with artesunate monotherapy and 7.5% (10/134) with SP. Even fewer could recall the correct number of days (31.3%) or correct number of tablets (29.9%) and how many times each day quinine should be administered (33.6%); combining all of these individual elements in to a composite indicator for knowledge of correct drug and dosing regimen for treatment of uncomplicated malaria in the first trimester, only 20.2% (27/134) of ANC providers demonstrated full knowledge (Table 5).

**Table 5:** Health worker knowledge of policy for treatment of uncomplicated malaria in the first trimester of pregnancy in Ashanti Region, Ghana (N = 134).

Indicator	N	%	95% CI
Proportion who know quinine is the correct drug for malaria treatment in first trimester	68	50.7	39.8, 61.6
Proportion who know the correct number of days for quinine treatment (7 days)	42	31.3	22.4, 42.0
Proportion who know the correct number of times quinine should be taken each day (3 times)	45	33.6	24.0, 44.7
Proportion who know the correct number of 600 mg quinine tablets that should be taken each time (1×600 mg)	40	29.9	21.7, 39.6
Proportion who know the correct dosing regimen of quinine to treat uncomplicated malaria during first trimester of pregnancy	27	20.1	13.2, 29.5

The only factor significantly associated with provider knowledge of correct quinine treatment during univariate log binomial regression was attendance at a workshop on malaria in pregnancy (RR: 2.51; 95% CI: 1.16,5.44; p = 0.02); the timing of this workshop was not significant (Table 6).

**Table 6:** Univariate predictors of composite first trimester malaria treatment, and second/third trimester malaria treatment knowledge indicators (N = 134 health workers).

		Knows full malaria treatment policy for 1^st^ trimester*				Knows full malaria treatment policy for 2^nd^ & 3^rd^ trimesters**			
Predictor	Sub-category	%	RR	95% CI	P value	%	RR	95% CI	P value
Age (years)	20–29	6.3	1.00		0.15	28.1	1.00		0.27
	30–39	21.4	3.43	0.89,13.2		42.9	1.52	0.66,3.51	
	40–49	31.3	5.00	1.19,21.0		25.0	0.89	0.44,1.79	
	50 or older	21.4	3.43	0.84,14.1		41.1	1.46	0.87,2.46	
Cadre	Midwife/nurse	26.9	1.00		0.24	40.3	1.00		0.55
	Nursing assistant	20.0	0.74	0.25,2.14		26.7	0.66	0.33,1.31	
	Community health nurse	9.7	0.36	0.11,1.18		29.0	0.72	0.39,1.34	
	Other	0	-	-		33.3	0.83	0.34,2.00	
Time at health facility	<1 year	21.9	1.00		0.16	34.4	1.00		0.95
	1–3 years	8.3	0.38	0.11,1.29		36.1	1.05	0.54,2.03	
	>3 years	25.8	1.18	0.55,2.53		33.3	0.97	0.51,1.86	
Time in current ANC	<1 year	18.4	1.00		0.35	36.8	1.00		0.96
	1–3 years	12.5	0.68	0.22, 2.06		32.5	0.88	0.49,1.58	
	4–9 years	27.5	1.49	0.68,3.29		32.5	0.88	0.45,1.73	
	>10 years	25.0	1.36	0.49,3.79		37.5	1.02	0.46,2.24	
Responsible for prescriptions	No	12.5	1.00		0.46	12.5	1.00		0.06
	Yes	21.2	1.69	0.41,6.94		37.3	2.98	0.93,9.53	
Attended malaria diagnosis workshop	No	11.1	1.00		0.11	25.0	1.00		0.18
	Yes	23.5	2.11	0.85,5.25		37.8	1.51	0.83,2.76	
Year of malaria diagnosis workshop	2009	29.7	1.00		0.55	48.6	1.00		0.16
	2008	23.5	0.79	0.30,2.06		35.3	0.73	0.33,1.58	
	2007 or before	18.2	0.61	0.25,1.48		29.5	0.61	0.36,1.02	
Attended MiP workshop	No	10.7	1.00		0.02	23.2	1.00		0.04
	Yes	26.9	2.51	1.16,5.44		42.3	1.82	1.02,3.24	
Year of MiP workshop	2009	31.4	1.00		0.59	42.9	1.00		0.48
	2008	28.6	0.91	0.37,2.23		52.4	1.22	0.64,2.32	
	2007 or before	18.2	0.58	0.20,1.71		31.8	0.74	0.34,1.62	

*Defined as the proportion that know the correct drug, dose and duration; not including restrictions to using quinine in 1^st^ trimester only for uncomplicated malaria.

**Defined as the proportion that know the correct drug, dose & duration to treat uncomplicated malaria during second/third trimesters of pregnancy, including restrictions on use of AS–AQ during first trimester.

When asked about the recommended treatment for uncomplicated malaria in the second and third trimesters of pregnancy, 78.4% (105/134) of providers correctly responded with the answer AS–AQ. More detailed questions followed about the appropriate dose and duration of AS–AQ treatment, regardless of whether the respondent had responded with AS–AQ as the recommended drug. A high proportion of providers could recall the correct duration and number of times AS–AQ should be taken each day (89.6%, [120/134] and 93.3%, [120/134], respectively). Knowledge of the correct number of AS and AQ tablets was lower (64.9% and 50.7%, respectively).

Combining all of these individual elements in to a composite indicator for correct drug and dosing regimen for treatment of uncomplicated malaria in the second or third trimesters, only 41.8% (56/134) of ANC providers demonstrated full knowledge (Table 5). The majority of respondents knew that AS–AQ should not be given in the first trimester (73.9%, 99/134); when this restriction was included with the full treatment indicator, 34.3% (46/134) of ANC providers demonstrated full knowledge (Table 7).

**Table 7:** Health worker knowledge of policy for treatment of uncomplicated malaria in the second and third trimesters of pregnancy in Ashanti Region, Ghana (N = 134).

Indicator	N	%	95% CI
Proportion who know that artesunate-amodiaquine (AS–AQ) is the correct drug for malaria treatment in second/third trimesters*	105	78.4	70.7, 84.5
Proportion who know that AS–AQ treatment should be given for 3 days*	120	89.6	81.2, 94.5
Proportion who know AS–AQ should be taken 2 times each day*	125	93.3	85.4, 97.1
Proportion who know that 2 artesunate (AS) tablets should be taken each time	87	64.9	55.3, 73.5
Proportion who know 2 amodiaquine (AQ) tablets should be taken each time	68	50.7	41.4, 60.1
Proportion who know 2AS and 2AQ tablets should be taken each time	66	49.3	39.8, 58.8
Proportion who reported AS–AQ should not be given in the first trimester	99	73.9	64.9, 81.3
Proportion who know the correct drug, dose & duration to treat uncomplicated malaria during second/third trimesters of pregnancy	56	41.8	32.2, 52.0
Proportion who know the correct drug, dose & duration to treat uncomplicated malaria during second/third trimesters of pregnancy, including restrictions on use of AS–AQ during the first trimester.	46	34.3	25.7, 44.1

*Note: the proportion of ANC staff that could report the correct duration of treatment with AS–AQ was higher than those that reported AS–AQ as the first-line drug for treatment of uncomplicated malaria in pregnancy; this is because questions on dose and duration of AS–AQ treatment for a pregnant woman in her second or third trimester were asked to all respondents, irrespective of their answer to the most appropriate drug.

Those responsible for writing prescriptions during ANC clinics (RR: 2.98; 95% CI: 0.93,9.53; p = 0.06) and providers who had attended a workshop on malaria in pregnancy (RR: 1.82; 95% CI: 1.02,3.24; p = 0.04) were more likely to know the correct treatment regimen of AS–AQ in the second or third trimesters; timing of this workshop was not significant (Table 6). No other variables were significantly associated with correct knowledge of treatment for uncomplicated malaria in the second or third trimesters. When these two variables were included in a multivariate model, workshop attendance retains borderline significance (RR: 1.70; 95% CI: 0.96,3.03; p = 0.07), whereas responsibility for prescriptions is no longer significantly associated with knowledge of AS–AQ treatment policy (RR: 2.64; 95% CI: 0.81,8.64; p = 0.11).

### Qualitative key informant interviews

#### Experience & perceptions of anti-malarial drugs for pregnant women

Overall, health worker perceptions on the use of different anti-malarial drugs during pregnancy supports the quantitative data in terms of believing that SP is for prevention and that AS–AQ and quinine are for treatment:

*“I would prefer the use of SP as a preventive treatment but if the patient has malaria, I would use the AS*–*AQ. The SP cannot treat malaria; it is only a preventive treatment and it becomes more effective if the person sleeps under insecticide treated nets and also, keep their environment clean.”* (Interview 501, Senior Nursing Officer)

Side effects to both SP and AS–AQ were mentioned but most ANC staff interviewed did not see these as inhibitory. For example, they considered that the common adverse effects associated with AS–AQ of weakness and dizziness can be avoided by telling a woman to eat properly before she takes the drugs; most reported reactions to SP relate to a woman being allergic to sulpha containing drugs, in which case the health worker would change the treatment. For example, in response to a question on how pregnant women feel about AS–AQ:

*“Some complain of weakness the first time they take it so they rush here to report that they feel weak. And we explain to them that, that is the nature of the drug so if they take the drug and do not eat well, they will feel weak but even then, the weakness lasts for just 2*–*3 days. They become alright if they are taught the right way to take the drug.”* (Interview 602, Nursing Assistant)

Chloroquine was spontaneously mentioned by a number of those interviewed in relation to the treatment of malaria in pregnancy with a preference for the current drugs (SP and AS–AQ) due to fewer side effects in pregnant women; likewise side effects and the long duration of the quinine treatment regimen were given as justification for their preference for AS–AQ.

##### Use of RDTs by ANC staff

Many midwives expressed support for the use of RDTs as these reduce reliance on microscopy which can only be performed by trained personnel and depends on electricity supply. Use of an RDT would also save women from queuing for long periods of time at the laboratory.

*“It will be better for us to use this [RDT] than going to the lab because there may be power cuts and unavailability of lab personnel in urgent situations especially on weekends. It is faster and can be used by the midwife for rapid results. It will also help the rural folks in their centres.”* (Interview 301, Midwife)

However, the almost universal negative consequence of the introduction of RDTs in to routine ANC was perceived to be the additional workload it would present, especially as free antenatal care has seen the numbers attending ANC increase since its introduction in July 2008.

On the other hand, the staff interviewed seemed motivated and confident that they could use RDTs with appropriate training and even if they did not receive support from additional staff then they would find coping mechanisms as ultimately looking after the pregnant women is their main objective. Task sharing between midwives (and the laboratory and dispensary) in completing their routine ANC activities already appears to be common. For example, after an initial health education talk for all attendees, ANC clinics in a number of facilities are arranged in to “stations” which each woman passes through to receive services such as palpation, blood pressure and weight measurements, history taking, IPTp and PMTCT. If RDTs were introduced then a number of respondents suggested that they could be incorporated in to this system:

*“The problem we face here is that the midwives are few but if more midwives are trained... Personally if it is fused with my work it won*'*t be a problem because wherever the pregnant woman finds herself the midwife will be there to help her and there wouldn*'*t be a problem of maternal death or premature delivery or even abortion. For me, it is good.”* (Interview 102, Midwife)

*“Division of labour... If we are many it will help us. If we are all trained in taking the samples so that whilst one [of us] is taking care of one [mother], another [of us] is taking care of another [mother]”* (Interview 203, Midwife)

##### Acceptability of IST versus SP-IPT

The general view of the midwives is that whatever misgivings pregnant women might have about either the drugs or repeat blood tests, they can be overcome by explaining and educating them that it is for the health of themselves and their babies.

It is also generally accepted that, regardless of opinions or experiences of pregnant women suffering adverse effects, SP is for prevention and AS–AQ is for treatment. However, perhaps reflecting on the nature of the role of ANC providers, when the IST strategy was summarised and they were directly asked which strategy they would prefer between IST and SP-IPT, more tended to favour SP-IPT stating the old adage that “prevention is better than cure”:

*“Fansidar [SP] is for preventive purposes. It is not used for treatment. It is the AS*–*AQ that is used to treat malaria. But because prevention is better than cure, I prefer the use of Fansidar for prevention.”* (Interview 302, Midwife)

This was not a universal view however, with a small number of midwives interviewed stating that they would prefer to test women to be sure that they had malaria before giving them “strong” drugs such as AS–AQ unnecessarily, and to make sure that they did not miss any other diagnosis.

*“You see no-one likes taking drugs and with these pregnant women, I think some of them are not even taking their routine drugs... The SP is for prevention, the person may ask why she should take it when she doesn*'*t have the MPs [malaria parasites]... Personally, I prefer the AS*–*AQ. The blood should be tested and if the woman has the MPs she will be given the AS*–*AQ”* (Interview 102, Midwife)

*“The AS*–*AQ is best for treatment. But I will only give the AS*–*AQ out if she has been diagnosed at the lab for MPs [malaria parasites].”* (Interview 201, Midwife)

It should be noted that some of the responses to this question suggest that not all of the interviewees fully understood the IST approach, despite working in facilities where the trial had taken place; they expressed concern that some women present too late with symptoms of malaria for effective treatment, hence their preference for “prophylactic” treatment with SP.

### Discussion

The quantitative and qualitative data presented here support the view that malaria is perceived as a serious problem for pregnant women in the Ashanti Region of Ghana.

Knowledge of most of the key components of the IPTp policy is extremely good with over 95% knowing that SP is the recommended drug, that women should receive three doses during their pregnancy, with an interval of a month between doses, and that SP-IPT should be given as directly observed therapy (DOT); 92% also knew that the first dose should be given after 16 weeks or quickening. Knowledge of the restrictions around the use of SP for IPT is less strong. For example, only around 50% could recall that SP-IPT should not be given in the first trimester, or ninth month. However, since most respondents knew the correct time to start SP-IPT, it is possible that this question was not fully understood, or perhaps the ANC personnel interviewed are not aware that the reason for timing of SP-IPT doses is due to the potential adverse consequences of taking SP in the first trimester of pregnancy. None of the respondents reported that SP-IPT is contraindicated if a woman is taking daily cotrimoxazole prophylaxis to prevent HIV-related opportunistic infections; this perhaps relates to the low HIV prevalence of 3.6% amongst pregnant women in Ghana [18] meaning that few pregnant women are likely to be taking prophylactic cotrimoxazole and so health worker experience of this is low.

Knowledge of the SP-IPT policy was stronger than that of the malaria treatment policies for pregnant women, particularly treatment with quinine for uncomplicated malaria in the first trimester. Knowledge of the timing restrictions for giving quinine for uncomplicated malaria was poor; in the case of AS–AQ, a bigger problem was in reporting the appropriate AQ dose. Findings from the qualitative study suggest this may be due to experience of adverse effects of AQ which leads to health workers giving lower doses (rather than not knowing the correct dose *per se*); health worker mistrust of AQ has been demonstrated elsewhere [19], although overall the respondents in this study in Ashanti Region observed that AS–AQ was effective in treating pregnant women with malaria and considered it to be generally acceptable, despite side effects in some women.

Lack of clarity around the timing and restrictions on the use of quinine for uncomplicated malaria may relate to the low numbers of women seen at ANC in their first trimester; in addition, quinine is also the recommended treatment for complicated malaria at any gestation. However, this lack of knowledge may simply be due to the fact that ANC staff in the majority of facilities in this study do not have responsibility for dispensing anti-malarial treatment; although 88% of respondents reported that they were responsible for writing prescriptions during ANC clinics, only 25% of facilities had AS–AQ available in their ANC department for dispensing by midwives, compared to the 70% that had SP available for dispensing by midwives. Quinine dispensing in the ANC was not specifically investigated. Responsibility for giving SP-IPT during ANC was a predictor of borderline statistical significance of full knowledge of SP-IPT policy; similarly for AS–AQ, those responsible for prescriptions during ANC were more likely to know the full second and third trimester treatment indicator. This was not the case for quinine; the reason for the discrepancy between AS–AQ and quinine is not clear. The qualitative analysis found that ANC staff may send pregnant women with suspected malaria for diagnostic tests and write a prescription for an anti-malarial if they have a confirmed diagnosis. However, the details of drug and dosage are the responsibility of dispensary staff. Perhaps personal experience of AS–AQ treatment compared to quinine is responsible for better knowledge of the treatment regimen.

The main predictor for success with all three knowledge indicators was attendance at a workshop on malaria in pregnancy. For the SP-IPT composite indicator, recent training was a factor, whereas for knowledge of treatment with quinine in the first trimester and AS–AQ in the second/third trimesters the timing of the workshop was not important. Unfortunately since the sampling unit was health facilities and there is likely to be some clustering on the knowledge outcomes within health facilities, the study was not powered to explore the multivariate effect of the potential predictors of knowledge. Nevertheless, the findings presented here suggest that training can be effective in improving knowledge of ANC staff on certain aspects of malaria such as the consequences of malaria for the pregnant woman and her baby, preventive measures and (to some extent) treatment. However, malaria treatment practice was not observed as part of the current study and although correct knowledge is essential it is not sufficient to assume translation to appropriate behaviour; indeed, several studies in different settings have shown quality of malaria case management is not significantly influenced by in-service training or guidelines [20], [21], [22], [23].

Similarly, despite the high knowledge of SP-IPT policy described here, the 2008 Ghana demographic and health survey (DHS) found that only 50.8% of women in Ashanti Region with a live birth in the preceding two years had received two or more doses of SP-IPT [12]. Routine data from the health facilities in this study show medians of 63%, 41% and 20% of ANC registrants in 2008 receiving one, two and three doses of SP-IPT, respectively. Low coverage of SP-IPT, especially the second and third doses is known to be of concern to the district and regional health authorities in Ashanti Region with late attendance by pregnant women often blamed for this. However, it is unlikely that this is the only reason for low SP-IPT coverage. For example, 21.3% (10/47) of the ANC clinics in this study which dispensed SP reported stock outs in the 6 months before the survey, in part attributed to delays in reimbursement of facilities that provide free ANC through the national health insurance scheme; additionally, only 58.2% (39/67) always had water freely available for DOT, matching challenges to SP-IPT implementation described for other settings [24], [25], [26].

The relatively low coverage of SP-IPT in Ashanti Region, despite the strong knowledge and acceptance of the strategy by health workers, supports the need for operational research to identify areas of weakness within the health system that need targeting by interventions. This is particularly relevant when considering introducing a new strategy. For example, if rising SP resistance means that SP-IPT is no longer effective, intermittent screening and treatment may be considered for roll-out as an alternative approach for MiP control. Maintaining stock of RDTs and AS–AQ will be vital if the efficacy of IST found under trial conditions is to translate to similar levels of effectiveness under operational conditions.

A key question is whether midwives would be able to deliver IST in the ANC. The first essential of IST is effective diagnosis of malaria. Microscopy services are available in 40% of all facilities in Ashanti but only around 20% of the lower level facilities have microscopes or conduct malaria blood slides. Therefore, if IST were to be introduced as a MiP intervention with diagnosis undertaken in the ANC, this would have to rely on RDTs.

The situation with regard to dispensing of anti-malarials is less clear. The vast majority (91.0%) of facilities have a dispensary and although there is some dispensing of drugs in other departments such as on the wards of hospitals and health centres, and in the ANC clinics, 30% of facilities do not keep SP in their ANC clinics for dispensing by midwives and almost 75% do not keep AS–AQ in the ANC. Therefore, it is uncertain whether women found to have malaria whilst attending an ANC clinic should be treated in the ANC or whether the woman should be sent with a prescription to the dispensary.

Other potential barriers to a change in strategy if it were to be implemented by ANC providers is the strong emphasis on prevention versus treatment amongst the midwives, reflecting their role as primary health care agents, and the worry about increased workload if they are expected to use RDTs and dispense ACT during ANC clinics. The concern of certain providers that some women present too late with symptoms of malaria for effective treatment underscored their preference for “prophylactic” treatment with SP. However, the principle of intermittent screening and treatment is that women would be tested with an RDT at key scheduled ANC visits, regardless of whether they reported malaria symptoms, so that asymptomatic malaria would also be identified and treated early. This misconception suggests that if IST were to be introduced, the details of the strategy would need to be very clearly explained to avoid resistance to the idea of treatment versus prevention. Nevertheless, the in-depth interviews with providers in the trial facilities revealed that there was little direct opposition to the IST strategy and many of the midwives related other stories of evidence influencing practice such as the change of malaria treatment policy from chloroquine to SP and then AS–AQ due to increasing drug resistance. This suggests that if research shows that SP is beginning to fail for use as IPT then an alternative strategy such as IST may be accepted, particularly if coupled with promotion of ITN use following the Ashanti IST trial protocol [6].

Similarly, it is likely that increased workload may not be as significant a barrier as the health workers suggest [27] with demonstrated task sharing and team work being important elements of day-to-day operation in the Ashanti Region health facilities surveyed. The main motivation of the health workers interviewed in-depth was the well being of the pregnant women under their care.

ANC staff already carry out a diverse range of tasks: they test for HIV, syphilis and urine protein using point-of-care devices. There is currently a strong advocacy for using point-of-care devices in health facilities for diagnosis. So another question now emerging is whether IST should be a stand-alone strategy for control of malaria in pregnancy or whether screening for other infections in pregnancy, such as sexually transmitted infections, could be linked to it.

In terms of delivering IST, the general view of the midwives is that whatever misgivings the pregnant women might have about either the drugs or repeat blood tests, they can be overcome by explaining and educating them that it is for the health of themselves and their babies. Interestingly, this correlates well with the findings of focus group discussions with mothers involved in the Ashanti IST trial for whom trust of ANC staff was found to be a more critical factor in the acceptance of either strategy than any particular characteristics of the strategies themselves [10]. Hence health worker support for any malaria in pregnancy intervention delivered through ANC is critical.

Cost effectiveness is an important element for decision-making in terms of any future policy change. In the case of IST this relates to both deciding whether to introduce the IST approach in place of SP-IPT, and also to the most efficient way that this strategy could be implemented. Economic modelling would be useful in determining whether the IST strategy is more cost effective as an ANC-based intervention in lower level facilities which do not have malaria microscopy when compared to hospitals. Costs data were collected alongside the original Ashanti IST trial and a cost effectiveness evaluation is currently being finalised and prepared for publication which will add further information to the debate for policy makers considering future options for MiP interventions. Further information on the costs of IST is being collected during the course of the multicentre trial currently underway in four countries in West Africa.

One potential limitation of this study is that the qualitative data are from facilities involved in the Ashanti IST trial. However, these two districts were purposefully not selected in the quantitative study. It is therefore possible that the in-depth opinions given by health workers in these two trial districts may be different to those who took part in the questionnaire survey. However, the two sources of data were intended to be complementary i.e. the quantitative data on MiP knowledge is representative of the majority of ANC providers in Ashanti region, whereas qualitative data on the potential barriers or facilitators to implementation of IST is drawn from providers with some exposure to the intervention and how it might fit with their usual responsibilities. Nevertheless, ANC providers in the qualitative study were not directly involved in delivering the trial interventions and their strength of opinion on the *status quo* suggests their knowledge and perceptions had not been very noticeably changed by the presence of the project team in their facilities so that it is not unreasonable to draw the two sources of data together.

#### Conclusion

If IST was considered by policy makers to be a viable and necessary alternative to SP-IPT and that it should be introduced and implemented as part of routine ANC activities, considerable attention would need to be paid to improving the knowledge and practices of ANC staff in relation to appropriate treatment of confirmed malaria in pregnancy. Similarly, it is possible that the strong belief in preventive measures such as IPTp and ITNs for malaria in pregnancy control may be an inhibitory factor for some ANC staff in shifting from IPTp to IST. However, this appears to reflect the effectiveness of MoH emphasis and training on preventive interventions and if similar attention were given to the IST strategy it may also see similar impact in terms of provider knowledge. In addition to improving health system factors such as a reliable supply chain, gaining the confidence and support of health workers that provide ANC services and deliver malaria interventions to pregnant women would be critical for successful implementation.
